# Comparative Studies on the Physicochemical and Volatile Flavour Properties of Traditional Deep Fried and Circulating-Air Fried Hairtail (*Trichiurus lepturus*)

**DOI:** 10.3390/foods11172710

**Published:** 2022-09-05

**Authors:** Yixuan Ding, Ting Zhou, Yueqin Liao, Huimin Lin, Shanggui Deng, Bin Zhang

**Affiliations:** 1 Zhejiang Provincial Key Laboratory of Health Risk Factors for Seafood, College of Food and Pharmacy, Zhejiang Ocean University, Zhoushan 316022, China; 2 Pisa Marine Graduate School, Zhejiang Ocean University, Zhoushan 316022, China

**Keywords:** hairtail, deep frying, air frying, HS-GC-IMS

## Abstract

The aim of this study is to investigate the effects of deep frying (DF) and air frying (AF) on the quality and flavour profile of hairtail (*Trichiurus lepturus*) fillets. The changes of some physicochemical indices such as moisture content, oil content, colour, thiobarbituric acid reactive substances (TBARS) and peroxide values (POV) in hairtail fillets were detected with increasing frying time. According to these physicochemical indices and sensory evaluation, deep frying for 7 min under 190 °C (DF7) and air frying for 24 min under 190 °C (AF24) were selected as samples for their great quality. The flavour fingerprint of hairtail (Raw, DF7, AF24) was developed and volatile compounds were investigated by HS-GC-IMS. A total of 28 volatile substances including aldehydes, alcohols, ketones and others were identified both in the DF7 and AF24 samples. There are differences in the aroma fingerprint between the DF7 and AF24 samples. DF was characterised by 2-Heptanone, (E)-2-Heptenal, 2-Pentyfuran and 1-Pentanol, AF was characterised by 2-methylbutanol, Ethyl methyl ketone-M and 3-hydroxy-2-butanone. These findings suggest that the aroma of hairtail fillets after DF7 and AF24 was significantly different and supply flavour information and practical applications of the fried hairtail fillets.

## 1. Introduction

The hairtail (*Trichiurus lepturus*), a common benthopelagic commercial marine fish in the eastern Pacific Ocean, is one of the most popular fish in Chinese cuisine for its appealing flavour and taste, as well as its high nutritional value, which includes plentiful essential amino acids and polyunsaturated fatty acids [1]. As one of the most commonly used processing methods, the thermal procedure not only inactivates and destroys microorganisms in the product by denaturising their proteins [2], but also ripens the food product by changing its flavour and texture [3] and it can be thermalised in a variety of ways, mainly including steaming and frying.

Fried hairtail fillets are appreciated by consumers for being crispy and delicious while their production process is simple. However, excessive consumption of high-calorie foods can cause obesity, high blood pressure and diabetes, all of which are risk factors for heart failure [4]. As a result, consumers are increasingly looking for more nutritious and safer food alternatives and researchers are focusing their efforts on developing new low-fat products [5]. Air frying is created to minimise these drawbacks. An air fryer makes use of a rapid heat generator at the top and high-speed hot air circulation technology to cook the food. This causes the fat and oils from the ingredients themselves to come out. Consumers get the same delicious cooking result as deep frying but without having to use extra cooking oil, one of its advantages is to reduce the cost of oil, reduce the emission of pollutants in the environment and save energy [6]. Many households believe that air frying is healthier than deep frying. In recent years, the majority of research on fried fish is concentrated on mass transfer, nutrient composition and the manufacture of low-fat fried fish fillets [7,8,9], whereas the research on the difference of flavour characteristics between deep frying (DF) and air frying (AF) fish fillets is rarely conducted. To make the basic aroma information clear and to provide a direction for improving flavour quality, we specifically investigate the differences in volatile odour compounds (VOCs) of fish fillets after DF and AF.

Aroma is a highly essential physicochemical component that impacts the flavour quality of fried hairtail fillets. HS-GC-IMS is a useful technique for separating and detecting volatiles quickly, which combines the high separation capacity of GC with the fast response of ion mobility spectrometry (IMS), thus becoming popular in the examination of food flavours. It can test for trace amounts and semivolatile substances in foods [4]. There is an increasing number of published instances of this technique’s applicability. It has been used to identify changes in volatile compounds in sea cucumber (*Apostichopus japonicus*) during soaking in seasonings [10] and to analyse the flavour formation during the production of Dezhou braised chicken [11]. The technique was also used to assess the characteristic aroma profile differences of coloured quinoas [12] and investigate the effect of aging on the characteristic flavour of Nanjing water-boiled salted duck [13]. 

In this experiment, hairtail fillets were fried by DF and AF, we investigated the physicochemical properties (moisture content, oil content, sensory evaluation and colour), POV and TBARS in the fried hairtail fillets and their odour characteristics were determined by HS-GC-IMS. The purpose of this study is to compare the impacts of different frying methods on the flavour attributes of hairtail fillets and to provide some basic knowledge for regulating and controlling the flavour quality of fried hairtail products.

## 2. Materials and Methods

### 2.1. Materials

Fresh hairtail were obtained from a fishery market (Zhoushan, China) and the hairtails, packaged in ice, were transported to the laboratory within 20 min. The average length of the hairtail was 60–65 cm and the average weight was 0.4–0.5 kg. Soybean oil (COFCO Co., Ltd., Beijing, China) was purchased from a local market (Zhoushan, China). Trichloroacetic acid (TCA), glacial acetic acid, sodium thiosulfate, 2-thiobarbituric acid, and ethanol were purchased from Nanjing Chemical Reagent Co., Ltd. (Nanjing, China).

### 2.2. Hairtail Processing and Treatments

The fish samples were washed with running water firstly. The viscera, head and tail were removed and then the fish were cut into fillets (6 cm in length). The process of deep frying (DF) of hairtail fillets was performed as follows. The heating temperature was measured using an infrared thermometer (AS842A, SMART SENSOR Co., Ltd. Dongguan, China). Soybean oil was heated to (190 ± 2) °C in a deep-fryer (DMS-DZL101D, Foshan Demashi Network Technology Co., Ltd., Foshan, China). In deep frying, the hairtail fillets were immersed in hot oil at a temperature of 190 ± 2 °C for 3, 5, 7 and 9 min, respectively. After frying and cooling to room temperature, the residual oil on the fried hairtail fillets was removed by using filter paper. In the air frying, the air-fryer (HD9741 Xinan Technology (Shenzhen) Co., Ltd., Shenzhen, China) was preheated for 5 min at 190 °C, then the hairtail fillets were placed the air chamber and fried at 190 °C for 12, 16, 20, 24 min, respectively.

### 2.3. Moisture Content, Oil Content, and Colour Determination

The moisture and oil content of hairtail fillets were measured according to the AOAC official methods 950.46 and 960.39, respectively [14]. The moisture content was measured using a direct drying method. The colour profile (including Lightness (L*), green-red chromaticity (a*), and blue-yellow chromaticity (b*)) of hairtail fillets was determined with a CS-210 precise colourimeter (Hangzhou CHNSpec Technology Co., Ltd., Hangzhou, China), and each sample was measured three times on each side in three separate locations, with three replicates for each condition.

### 2.4. Peroxide Values (POV) and Thiobarbituric Acid Reactive Substance (TBARS) Determination

The peroxide value (POV) of hairtail fillets was measured by the method of ISO 3960:2017. A 50 g sample was mixed with 150 mL petroleum ether (boiling point of 30 °C–60 °C) overnight (37 °C) and then filtered, the filtrate was heated to a constant weight later. Then, 2 g of extracted lipids was mixed with 30 mL of chloroform-glacial acetic acid and 1 mL of saturated potassium iodide solution, and reaction mixture was left in the dark for 3 min. The reaction mixture was titrated with a solution containing 100 mL of water, 1 mL starch indicator, and 2 mmol/L sodium thiosulfate solution until the blue colour of the reaction mixture disappeared. The value of thiobarbituric acid reactive substances (TBARS) was determined according to Wang et al. [15]. The results were expressed as mg malondialdehyde (MDA) per kg (mg MDA/kg) of muscle.

### 2.5. Sensory Evaluation

Sensory scores of fried (DF and AF) hairtail fillets were evaluated at room temperature by ten professionally trained panelists from graduate students (five females and five males). The sensory attributes used were colour, texture, odour, taste and overall acceptability, which were developed and defined in accordance to the Chinese Stantard GB 37062, 2018. Samples were distributed at random onto plates with ten number codes (five DF samples and five AF samples) and a 10-point scale was used. The lexicon of sensory attributes is shown in Table S1.

### 2.6. Free Fatty Acids Determination

Free fatty acid (FFA) content of lipid extract was determined according to the method of Hu et al. [16]. Oil (15 mg) was dissolved in 2 mL of hexane and mixed with 100 µL methanol NaOH (1 mol/L) for esterification, followed by analysis with an Agilent 7890A. Agilent 7890A (GC-MS) was equipped with a flame ionisation detector and HP-5MS (30 m × 0.25 mm × 0.25 µm, Agilent Technologies Co., Ltd., Palo Alto, CA, USA). The GC oven temperature was kept at 50 °C for 1 min and programmed to 188 °C at a rate of 2 °C/min which was held for 9 min, then programmed to 240 °C at the same rate and kept constant for 5 min. The fatty acid content was determined by comparing the area of various analysed fatty acids with the area of a fixed concentration of internal standard.

### 2.7. Headspace Gas Chromatography-Ion Mobility Spectrometer (HS-GC-IMS) Analysis

The change of volatile profiles of hairtail fillets after frying was investigated by using headspace gas chromatography-ion mobility spectrometry (HS-GC-IMS) (FlavourSpec^®^, G.A.S., Dortmund, Germany) [10]. First, 2.00 g of minced sample was transferred to a 20.00 mL vial and incubated in an autosampler unit (CTC-PAL, CTC Analytics AG, Zwingen, Switzerland) at 60 °C (500 rpm for 20 min). Then, a gas sample of 500 µL was taken from the headspace and injected into the GC injector. The chromatographic conditions were as follows: MXT-5 capillary column (15 m × 0.53 mm × 1 μm), column temperature 60 °C, operating time 25 min, carrier gas N_2_ (purity ≥ 99.999%). The conditions of the analysis were as follows: 0–2 min, 2 mL/min; 2–10 min, 2–10 mL/min; 10–20 min, 10–100 mL/min; 20–25 min, 100–150 mL/min to stop. IMS temperature was 45 °C, purity N_2_ ≥ 99.999%. The drift gas flow was set to 150 mL/min. Analysis was carried out using the resulting difference maps, fingerprinting, and principal component analysis (PCA) maps. All results were measured in triplicates. To calculate the retention index (RI) of VOCs, the ketone C4–C9 (Sinopharm Chemical Reagent Beijing Co., Ltd., Beijing, China) was used as an external reference. VOCs were identified by comparing the RI and the drift time of a standard in the GC-IMS collection. The peak area signal intensity calculated using LAV software was utilised to compare the concentrations of volatile compounds quantitatively.

### 2.8. Data Analysis

All graphs in this study were created using Origin 2021 (Origin-Lab Corp, Northampton, MA, USA), and all data were analysed statistically using SPSS 21 (Chicago, IL, USA), The data were analysed using analysis of variance (ANOVA) and bivariate correlation analysis, and are presented as mean ± SD. Statistically significant differences between groups were determined by Duncan’s multiple range test at *p* < 0.05. PCA was performed with the Dynamic PCA plug-in (G.A.S., Dortmund, Germany). SPSS 21 and Origin 2021 were used for correlation analysis and visualisation of any differences and between samples.

## 3. Results and Discussion

### 3.1. Physicochemical Property Analysis

A key quality criterion for fried products is moisture content, which provides a crisp exterior and the proper amount of moisture in the interior to entice consumers. Table 1 shows the moisture content changes of fried hairtail fillets at different times. As shown in Table 1, the moisture content was reduced from 79.37% to 63.94% via DF for 9 min and from 79.37% to 65.61% through AF for 24 min, which could be explained by the difference in frying time. Longer frying times may result in more moisture loss under the same frying temperature. During the initial phases of the frying process, moisture content decreases at a far larger pace than it does later on, regardless of whether DF or AF. Since increasing the frying time causes the surface temperature of the hairtail fillets to rise rapidly, which could cause a dried layer to form [17]. It may also assist to maintain the internal moisture in the food preserved during the frying process [18]. Besides, as a result of the faster thermal transfer by oil than air and the capillary action of hot oil [19], DF reduced the moisture content of hairtail fillets faster than AF.

Oil content is one of the most critical aspects in determining the quality of fried products and consumer preferences. The change of oil content in fried hairtail fillets was shown in Table 1. DF had a much higher oil content than raw samples (13.28%) (*p* < 0.05), whereas AF had a substantially lower oil level (*p* < 0.05). This trend can be attributed to the fact that the oil of hairtail fillets melted and evaporated with the extension of AF time [20] without any added oil, its own oil partially spilled into the air-fryer’s bottom or was removed by the hot air circulating within the appliance [21]. Compared with the AF, the oil in DF hairtail fillets is mostly derived from frying oil, and the oil can further penetrate into the interior of the flesh of the hairtail due to moisture evaporation. As shown in Table 1, AF hairtail fillets resulted in a gradual increase in oil content because a substantial amount of oil droplets in the hot air were reabsorbed by hairtail fillets. Oil droplet buildup in the air fryer may increase slightly later [21].

**Figure 1:**
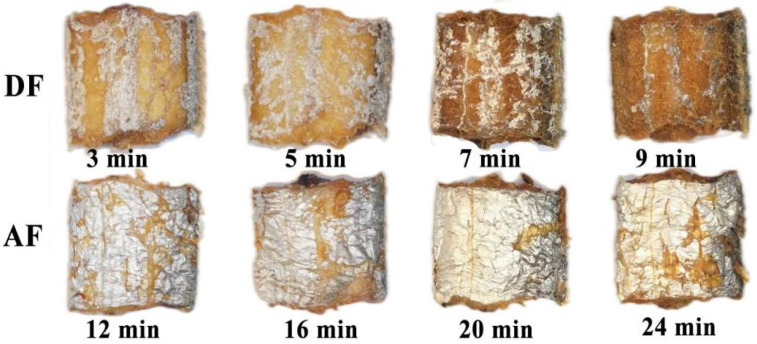
Photos of DF and AF at different thermal time.

Colour is an important index of quality and appearance evaluation of fried products that can attract consumers with bright colour. The colour changes (L*, a* and b* values) of the hairtail fillets during DF and AF with different thermal times are shown in Figure 1 and Table 1. Before frying, raw hairtail fillets showed L* = 87.53, a* = −4.20 and b* = −1.44. As an important parameter of the brightness of fried food [22], the L* value of fried hairtail fillets in DF decreased significantly (*p* < 0.05) with the frying time because the black compounds formed through the Maillard reaction [23] and moisture content release decrease the reflection of light as well [24]. However, there was no significant change in the L* value of fried hairtail fillets during AF. This trend can be attributed to the fact that AF preserves the fish skin of hairtail fillets well, which makes up for the serious loss of hairtail fillets fish skin after frying. In addition, the a* and b* values of DF hairtail fillets showed an increasing trend, because the various intermediates or final products of the Maillard reaction form green and yellow substances. Some studies have shown that the a* value has a direct correlation with the generation of the carcinogenic compound acrylamide in fried foods at relatively high temperatures [25]. the b* values of AF hairtail fillets decreased gradually with frying time, which resulted from the fat contained in the hairtail fillets melting at high temperatures [21] and partially adhering to the surface of the hairtail fillets during AF.

### 3.2. POV and TBARS Analysis

**Figure 2:**
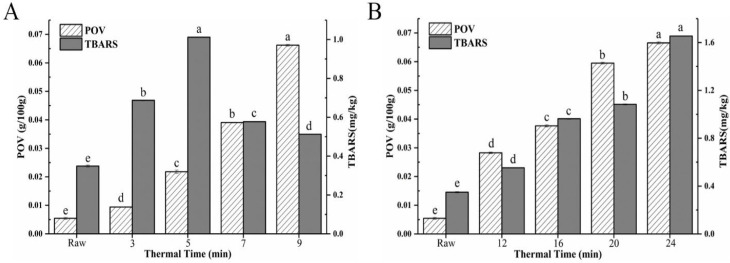
(**A**) POV value and TBARs of DF hairtail fillets. (**B**) POV value and TBARs of AF hairtail fillets. Different letters within each substrate indicate significant difference (*p* < 0.05).

Lipid oxidation produces a variety of flavour precursors that are involved in the peptide thermal degradation and the Maillard reaction under different processing conditions [26], which can be evaluated by the POV and TBARS. POV is usually used as the index of primary lipid oxidation-derived products and TBARS can reflect the content of secondary oxidation products aldehyde (MDA). Figure 2 showed that the POV value of raw hairtail fillets was 0.005 g/100 g and the TBARS value was 0.35 mg·kg^−1^. The POV value of DF and AF were higher than the raw hairtail fillets and POV value showed a significant elevating trend with the extension of frying time (*p* < 0.05). When the thermal time was prolonged from 3 min to 9 min, the POV of DF increased progressively from 0.009 g/100 g to 0.066 g/100 g and the POV of AF increased from 0.028 g/100 g to 0.067 g/100 g with the rise up of thermal time from 12 min to 24 min, which indicated increasing the heating temperature could substantially promote the formation of lipid peroxides and aldehydes (*p* < 0.05). Hence, the TBARS value of fried hairtail fillets also increased. As shown in Figure 2B, the rising rate of MDA in DF hairtail fillets was significantly higher than that in AF. Wu et al. [27] had shown that the high temperature and oil content on the surface of fried hairtail fillets enhanced the MDA content. Therefore, the MDA content of DF samples not only came from fried hairtail fillets itself but also from the oil, and the MDA content of AF samples only came from fried hairtail fillets itself. However, the TBARS value of DF began to decrease when the thermal time reached 5 min, because unsaturated aldehydes, binary aldehydes, and other active carbonyl compounds produced by fat oxidation can participate in the advanced stage of the Maillard reaction, and lipid peroxidation product MDA was likely to react with protein and amino acids, and thereby resulted in the loss of MDA and other carbonyl compounds that can react with TBA [28].

### 3.3. Sensory Evaluation

**Figure 3:**
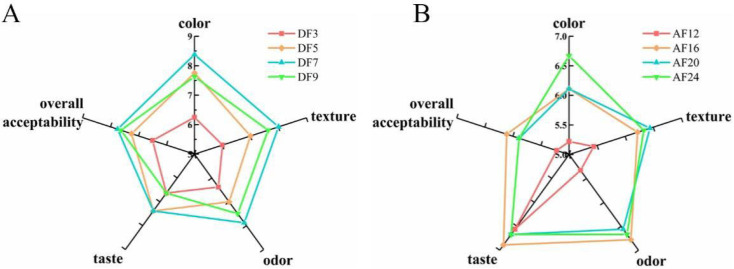
Radar chart of sensory scores for different fried hairtail fillets. (**A**) Radar chart of sensory scores of deep frying. (**B**) Radar chart of sensory scores of air frying. DF3, deep frying for 3 min; DF5, deep frying for 5 min; DF7, deep frying for 7 min; DF9, deep frying for 9 min; AF12, air frying for 12 min; AF16, air frying for 16 min; AF20, air frying for 20 min; AF24, air frying for 24 min.

Sensory evaluation is critical in determining the acceptance and preference of various fried products. The sensory scores of the fried hairtail fillets during DF (Figure 3A) and AF (Figure 3B) are shown in Figure 3. After DF for 3, 5, 7, 9 min and AF for 12, 16, 20, 24 min, the score of colour ranged from 6.25 to 8.38 and 5.22 to 6.67, respectively. The score of texture ranged from 6.00 to 8.00 and 5.44 to 6.44, respectively. The score of odour ranged from 6.38 to 7.88 and 5.33 to 6.78, respectively. The score of taste ranged from 6.63 to 7.38 and 6.56 to 6.89, respectively, while the score of overall acceptability ranged from 6.50 to 7.63 and 5.11 to 5.89, respectively. All these figures suggested that frying time had a significant effect on the quality hairtail fillets. In terms of colour, the hairtail fillets become golder and browner with increasing DF time, however, the AF hairtail fillets showed no substantial change. The texture scores elevated gradually with increasing frying time, which might be related to consumers preferring crunchy and chewy products [6]. With regard to odour and taste, the scores of DF hairtail fillets showed a sharp increase, since the DF samples gave a characteristic fried smell and flavour, whereas the AF time had no significant effect on it. In conclusion, when the DF time was 7 min, the fried fillets had a golden colour, crispy texture and rich flavour of fried food. When the AF time was 24 min, the sides of AF fillets were relatively bright and slightly golden, the skin was relatively intact, no greasy feeling, and the taste was moderate. The volatile odour compounds (VOCs) of hairtail fillets treated with these two frying process parameters (DF 190 °C for 7 min and AF 190 °C for 24 min) were compared and studied afterward.

### 3.4. Headspace Gas Chromatography-Ion Mobility Spectrometer (HS-GC-IMS)

**Figure 4:**
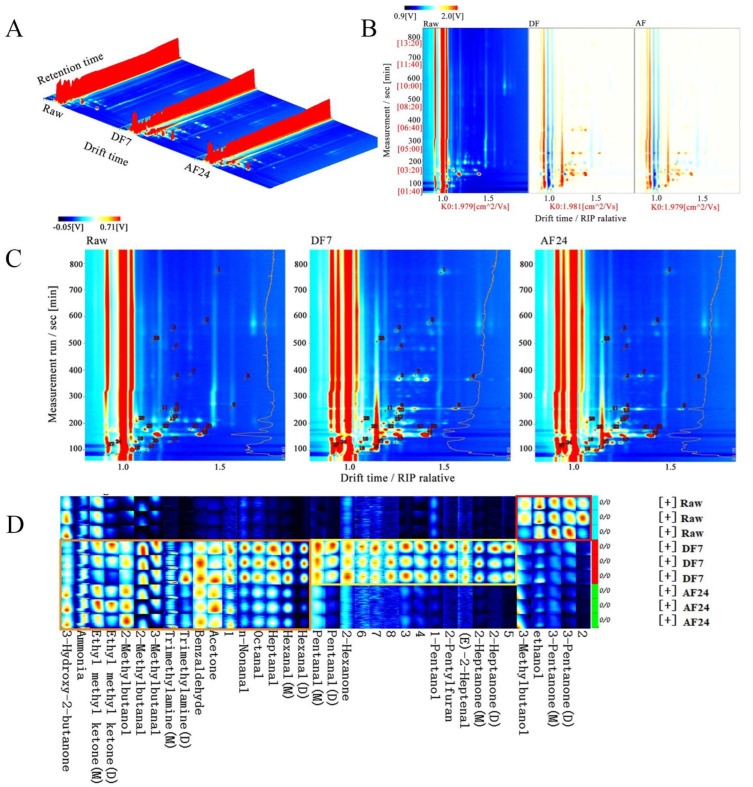
(**A**) Three-dimensional topographic of VOCs. (**B**) Comparison of the difference spectrum of volatile components. (**C**) Qualitative spectrum of volatile components. (**D**) Fingerprint comparison of volatile organic compounds (VOCs) of hairtail fillets in different frying methods determined by HS-GC-IMS. Raw, untreated; DF7, deep frying for 7 min; AF24, air frying for 24 min; Raw, untreated; DF7, deep frying for 7 min; AF24, air frying for 24 min.

The HS-GC-IMS approach is used to obtain global IMS information from the samples, and the topographic plot of the HS-GC-IMS fingerprinting was used for an intuitive comparison of the VOCs in DF7 and AF24 hairtail fillets. Figure 4A depicts the 3D spectra of volatile chemicals in DF7 and AF24 hairtail fillets. The X-axis represents ion migration time for qualitative analysis, the Y-axis represents VOCs retention time in the gas chromatograph, and the Z-axis represents peak height for relative quantification. As shown in Figure 4A compared with the Raw group, the signal strengths of VOCs in DF7 and AF24 samples were significantly different, and the kinds had also altered. Most VOCs have grown in concentration after the frying process, whereas others have decreased. Moreover, many new signal points appeared.

However, the 3D topography map was somewhat crude, which was not immediately apparent from the inspection of the data. Figure 4B shows a top view of GC-IMS 3D topography, with the Raw group serving as a reference from which all other treatment groups were subtracted, with the original background colour having been faded to white throughout the deduction process. The signal intensities of the substances were indicated by shades of colour variations. When the VOCs content was higher than that of Raw samples, it was indicated in red. On the contrary, it was indicated in blue. The content of some compounds with relative DT of 1.0–1.5 increased after DF7 and AF24. More red spots could be observed in DF7 and AF24 hairtail fillets than in the Raw samples, which can be attributed to various chemical reactions, such as polymerisation, hydrolysis, lipid oxidation, sugar dehydration, Maillard reaction and protein denaturation [29].

The locations of volatile odourants in the GC-IMS map are shown in Figure 4C. As shown in Figure 4C and Table 2, a total of 28 typical target compounds (including monomers and partial dimers) from topographic plots were identified by the GC-IMS NIST database, including 11 aldehydes, 9 ketones, 4 alcohols and 4 others. The flavour compounds were detected by GC-IMS and the fingerprint spectra data were analysed in Laboratory Analytical Viewer software (Figure 4D). In the red rectangle-labeled area, the content of 3-Pentanone, ethanol and 3-Methylbutanal in raw samples was significantly higher than the fried hairtail fillets after frying. In the yellow rectangle-labeled area, the content of 2-Heptanone, (E)-2-Heptenal, 2-Pentyl furan and 1-Pentanol in the hairtail fillets after DF7 surged (*p* < 0.05) but there was no significant change after AF24. In the orange rectangle-labeled area, most of the VOCs increased after frying. The aroma and taste of food are the most common and direct factors influencing consumers’ desire to purchase and evaluate the food as acceptable or unsuitable [30].

In light of previous research, straight-chain aliphatic aldehydes, alcohols, ketones, and certain hydrocarbons were most likely formed by the heat oxidative degradation of unsaturated fatty acids and lipids [11], while nitrogen compounds, such as 2-Pentyl furan came from Maillard reactions and Strecker degradation [31]. As shown in Figure 4C and Table 2, most of the VOCs in DF7 hairtail fillets were higher than those in AF24. In addition to the hairtail fillets themselves, most of the fat in DF7 hairtail fillets came from the frying oil, which might cause the higher VOCs. A previous study [32] reported that the amount and composition of fat were associated with the flavour of meat, with fish having a lower fat content and a less volatile flavour than other meats. The fried flavour is primarily made up of volatile compounds such as oily, fruity, grassy, buttery, nutty, and fishy [33]. Aldehydes are the main products of lipid oxidation and amino acid degradation [34], which are usually considered to be the primary flavour contributors to fish meat products due to their low thresholds and higher concentrations [35]. In these samples, nine aldehydes were detected, including n-Nonanal, Octanal, 2-heptenal (E), Heptanal, Hexanal, 2-methylbutanal, 3-Methylbutanal, Pentanal and benzaldehyde. Moreover, the dimers of Hexanal and Pentanal were also detected. The signal level of most aldehydes in DF7 hairtail fillets was substantially brighter than in air-fried hairtail fillets, according to the test results (Figure 4D). Hexanal and heptanal are mostly formed through the oxidation of linoleic acid and arachidonic acid, respectively [36]. The oxidation of oleic acid produces octanal, which has a citrus-like odour [37] and pentanal has a cheesy smell [38]. N-nonanal gives meat products a pleasant flavour (flower, orange, and grassy) [39]. After DF7 and AF24, the amount of short-chain (C < 10) aldehydes in fried hairtail fillets increased to various degrees. Except for Benzaldehyde, the content of aldehydes in DF7 hairtail fillets was significantly higher than AF24 samples. According to a prior study, short-chain aldehydes generally have a “fresh, grassy, green” aroma, and when carbon atoms add up, they might create the sensation of being “greasy and fatty” [40]. 2/3-methylbutanal have cheese [41] and malt aromas [42], which are formed mostly from the Strecker degradation of isoleucine/leucine during the Maillard reaction, contributed to the DF7 odour of the majority of fried products [43]. Benzaldehyde was the only aromatic aldehyde observed in fried hairtail fillet samples, giving products an almond and nut flavour; this was produced by Strecker degradation of phenylalanine or a linolenic acid [44].

Alcohols are formed by sugar metabolism, lipid oxidation, decarboxylation and dehydrogenation of amino acids [45], and some may be produced by the reduction of aldehydes. Generally, the threshold value of alcohols was higher, having a synergistic influence on the formation of flavour [46], while contributing less to the overall taste of food. In this study, alcohols in fried hairtail fillets were found to mainly include 1-pentanol, ethanol, 2-methylbutanol and 3-methylbutanol. After DF7 and air-frying, the signal intensity of ethanol fell dramatically (*p* < 0.05), owing to the strong boiling generated by the frying treatment, which increased the loss of volatile components [29]. The alcohol 2-methylbutanol is derived from isoleucine [47], while the content of it in fried hairtail fillets was considerably higher than that in the raw samples, and 3-methylbutanol smells like bananas and pears [48]. The level of 1-pentanol in DF7 hairtail fillets was significantly higher than the raw samples. This could be due to the large contribution of liquor flavour substances in the DF7 hairtail fillets.

Ketones are formed as a result of the heat oxidation and degradation of unsaturated fatty acids, as well as the Maillard reaction. Their thresholds are low, which has a modifying influence on flavour and frequently produces a fragrant scent [26]. As shown in Table 2 and Figure 4D, the content of ketones including Ethyl methyl ketone (M) and 3-Pentanone decreased significantly after DF7 and AF24 and 3-hydroxy-2-butanone with a pleasant cream flavour plunged after DF7, which might contribute to that as carbonyl compounds, ketones can react with amino acids, peptides, proteins, and other substances [44]. Furthermore, vastly different (*p* < 0.05) content of 2-heptanone was detected after DF7, however, no significant differences in 2-heptanone were observed between the raw samples and the AF24 samples. The present work showed that 2-heptanone degraded from linoleic acid and imparted products with a blue cheese flavour [49].

Furan is one of heterocyclic compounds for meat flavour that can be produced by Maillard reaction, lipid oxidation and thermal degradation [31]. This compound was the main flavour substance produced in the fried hairtail fillets by DF7 and AF24. No tremendous difference in the content of 2-Pentyl furan was observed between the raw group and AF24 group, however, the 2-Pentyl furan content in the DF7 group was greatly improved.

### 3.5. Similarity Analysis of Fingerprint-Based on PCA

**Figure 5:**
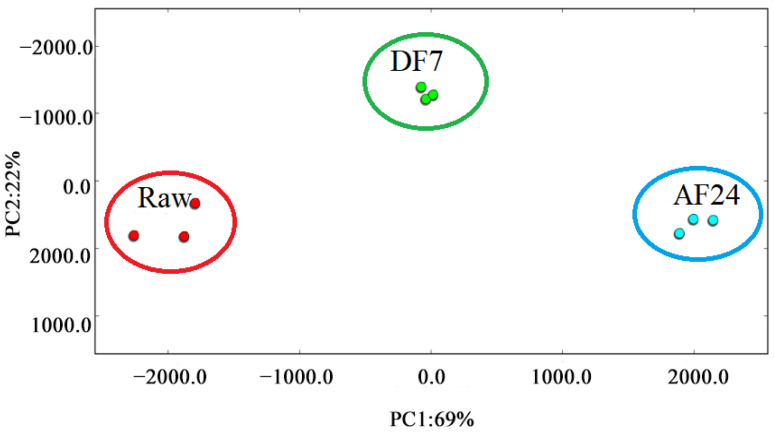
PCA based on the signal intensity obtained with hairtail fillets in different frying methods. Raw, untreated; DF7, deep fried for 7 min; AF24, air fried for 24 min.

PCA is a data-simplifying technique that reduces a large number of interrelated original variables to a small number of orthogonal principal component variables. A series of observations of possibly correlated variables are transformed into linearly uncorrelated variables, known as principal components, using an orthogonal transformation [50]. In an attempt to further understand the difference in the volatile profile among three samples, a total of 27 substantially different volatiles among samples were used for the PCA (Figure 5). The contribution rate of PC1 was 69%, the contribution rate of PC2 was 22%, and the cumulative contribution rate of the first two principal components was 91%, suggesting that the commonalities between various samples were sufficiently explained [51]. As shown in Figure 5, Raw, DF7 and AF24 were located in different places were respectively far away from each other. It indicated significant differences of volatile profiles present in different samples. This result is consistent with Table 2. Obviously (*p* < 0.05) different relative content of individual volatile compounds was present in different samples.

### 3.6. Correlation of Characteristic Volatile Flavour Compounds with FFA in Fried Hairtail Fillets

**Figure 6:**
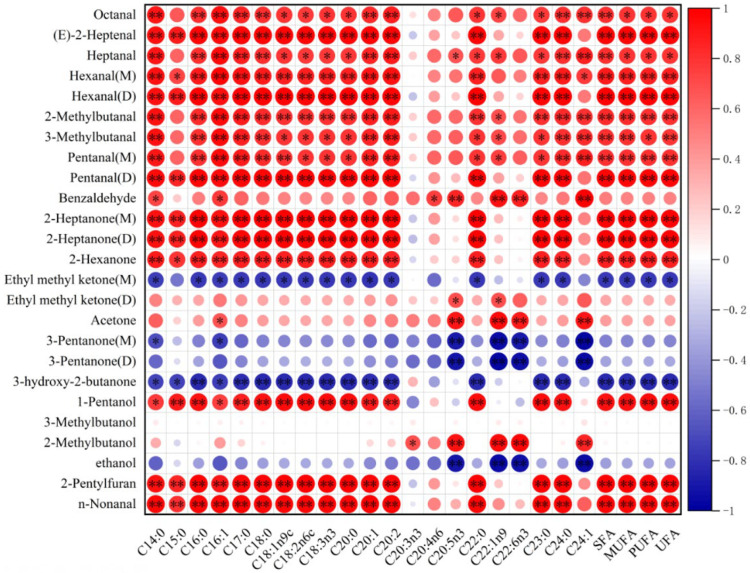
Correlation analysis of the free fatty acids and the volatile compounds in frying hairtail fillets. Asterisks stand for significant differences: * *p* < 0.05, ** *p* < 0.01.

Free fatty acids (FFAs) are the main precursor of volatile compounds in muscle. In this experiment, the correlation between volatile compounds and free fatty acids (Table S2) in fried hairtail fillets was assessed, and the data were visualised. As shown in Figure 6, most of the characteristic aroma components including aldehydes, ketones and alcohols were positively correlated with FFAs, while some ketones such as Ethyl methyl ketone (M) and 3-hydroxy-2-butanone were negatively correlated with most FFAs (*p* < 0.05). Furan was also affected by FFAs. Previous studies [52,53] had shown that there is a close correlation between furans and PUFA, which was in harmony with this study. The major products of fatty acid degradation are aldehydes and ketones [54], which are generated primarily through the oxidative decomposition of unsaturated fatty acids such as oleic acid, linoleic acid, and arachidonic acid. In this study, most of the ketones and aldehydes and partial alcohols show a high correlation with C18: 1, C18: 2 and C20: 4. Moreover, for some aldehydes such as 3-Methylbutanal, MUFA presented more effects on it than PUFA did. The only aromatic aldehyde found in fried hairtail fillets was benzaldehyde, and there was a positive correlation between it and C16: 1, C18: 1, C18: 2, C20: 2, and C20: 4 (*p* < 0.05).

## 4. Conclusions

This study showed the difference of quality and volatile flavour compounds in hairtail fillets after deep frying and air frying. The results showed that the moisture content decreased while the oil content increased in AF and DF with frying time and the colour of AF samples was similar to the raw for preserving the fish skin of hairtail fillets well, meanwhile, the TBARS content in DF showed a trend of increasing first and then decreasing, According to these physicochemical indices and sensory evaluation, the optimal frying times for deep frying and air frying were 190 °C for 7 min and 24 min, respectively. A total of 28 flavour compounds were detected by HS-GC-IMS, including aldehydes, ketones, alcohols and furans. The aromas of hairtail fillets after DF7 and AF24 were significantly different and the content of 2-Pentylfuran was higher in the DF7 samples. Through PCA analysis and correlation analysis, VOC samples varied greatly between DF7 and AF24, and many VOCs were potentially associated with specific FFAs mainly including oleic acid, linoleic acid and γ-linolenic acid. In conclusion, this study provided a reference for understanding the changes of flavour of fried fish fillets after DF and AF at different times.

## Figures and Tables

**Table 1:** Moisture, oil contents and colour of fried hairtail fillets.

	Thermal Time (min)	Moisture (%)	Oil (% db)	L*	a*	b*
Raw hairtail fillets	0	79.37 ± 0.20 ^aA^	13.28 ± 0.55 ^bA^	87.53 ± 0.80 ^aA^	−4.20 ± 0.37 ^cB^	−1.44 ± 0.09 ^cC^
DF	3	72.88 ± 2.55 ^b^	51.14 ± 0.13 ^a^	50.63 ± 0.55 ^b^	2.97 ± 1.01 ^b^	25.31 ± 2.43 ^a^
	5	70.69 ± 0.51 ^b^	51.90 ± 0.12 ^a^	47.17 ± 0.46 ^c^	2.34 ± 1.02 ^b^	18.92 ± 0.85 ^b^
	7	66.28 ± 0.61 ^c^	51.68 ± 0.22 ^a^	32.15 ± 2.26 ^e^	8.10 ± 1.57 ^a^	21.42 ± 1.82 ^a^
	9	63.94 ± 1.30 ^c^	51.79 ± 0.39 ^a^	38.06 ± 0.90 ^d^	8.04 ± 2.71 ^a^	18.83 ± 1.49 ^b^
AF	12	68.93 ± 2.79 ^B^	8.27 ± 0.23 ^D^	76.17 ± 14.43 ^B^	−5.26 ± 1.01 ^C^	3.51 ± 0.03 ^B^
	16	67.61 ± 1.26 ^B^	8.78 ± 0.29 ^CD^	80.85 ± 0.61 ^B^	−3.62 ± 0.91 ^B^	3.31 ± 0.16 ^B^
	20	67.15 ± 0.36 ^B^	9.37 ± 0.16 ^C^	76.98 ± 1.05 ^B^	−1.39 ± 0.30 ^A^	8.90 ± 0.62 ^A^
	24	65.61 ± 0.98 ^B^	10.78 ± 0.24 ^B^	78.40 ± 0.74 ^B^	−3.37 ± 0.46 ^B^	8.89 ± 0.64 ^A^

The different lower letters and upper letters in the same column represent a significant difference with *p* < 0.05 among each group. db: dry basis.

**Table 2:** The volatile components and relative contents of hairtail fillets in different frying methods. Data are represented as a mean ± standard deviation of three replicates. mean values with different lower-case letter in the same row correspond to significant differences at *p* < 0.05. Raw, untreated; DF7, deep frying for 7 min; AF24, air frying for 24 min.

No.	Compound	CAS	Formula	MW	RI	Rt	Dt	Volume (a.u)		
								Raw	DF7	AF24
1	n-Nonanal	C124196	C_9_H_18_O	142.2	1102.7	764.73	1.48198	145.62 ± 11.33 c	360.97 ± 15.49 a	197.30 ± 11.99 b
2	Octanal	C124130	C_8_H_16_O	128.2	1012.0	576.645	1.42008	52.17 ± 5.32 c	147.11 ± 19.97 a	108.37 ± 10.52 b
3	2-Pentylfuran	C3777693	C_9_H_14_O	138.2	995.7	547.771	1.25009	41.30 ± 0.57 b	114.31 ± 8.77 a	40.54 ± 6.82 b
4	2-heptenal (E)	C18829555	C_7_H_12_O	112.2	961.6	480.513	1.25897	42.38 ± 6.70 b	99.89 ± 5.86 a	50.87 ± 4.84 b
5	2-heptanone-M	C110430	C_7_H_14_O	114.2	892.6	368.45	1.26414	115.42 ± 0.94 b	707.31 ± 46.16 a	148.87 ± 8.59 b
6	2-heptanone-D	C110430	C_7_H_14_O	114.2	890.6	365.813	1.63386	19.55 ± 3.53 b	135.38 ± 18.85 a	21.55 ± 0.67 b
7	Heptanal	C111717	C_7_H_14_O	114.2	902.6	382.952	1.34946	31.46 ± 0.67 c	195.09 ± 13.92 a	134.92 ± 7.86 b
8	Hexanal-M	C66251	C_6_H_12_O	100.2	791.6	256.533	1.26397	99.32 ± 15.56 c	1067.04 ± 48.29 a	571.11 ± 102.92 b
9	Hexanal-D	C66251	C_6_H_12_O	100.2	792.8	257.59	1.55965	68.97 ± 4.61 b	578.51 ± 49.41 a	143.91 ± 51.09 b
10	1-pentanol	C71410	C_5_H_12_O	88.1	772.9	239.198	1.25178	176.01 ± 17.38 b	353.77 ± 18.00 a	98.21 ± 20.99 c
11	2-Hexanone	C591786	C_6_H_12_O	100.2	781.8	247.443	1.19285	51.17 ± 3.30 b	65.71 ± 7.54 a	51.69 ± 0.21 b
12	3-methylbutanol	C123513	C_5_H_12_O	88.1	743.5	213.606	1.24616	147.37 ± 34.93 a	150.65 ± 8.54 a	151.27 ± 6.05 a
13	2-methylbutanol	C137326	C_5_H_12_O	88.1	731.8	204.281	1.23224	81.09 ± 22.83 c	187.31 ± 29.44 b	287.64 ± 11.88 a
14	2-methylbutanal	C96173	C_5_H_10_O	86.1	669.8	163.705	1.39831	537.92 ± 289.44 c	2531.89 ± 355.15 a	1584.82 ± 304.31 b
15	3-Methylbutanal	C590863	C_5_H_10_O	86.1	648.2	153.624	1.39831	649.16 ± 313.46 c	2728.27 ± 381.04 a	1815.76 ± 303.20 b
16	Ethyl methyl ketone-M	C78933	C_4_H_8_O	72.1	592.1	130.185	1.06525	603.74 ± 8.10 a	444.85 ± 92.32 b	590.96 ± 89.19 ab
17	Ethyl methyl ketone-D	C78933	C_4_H_8_O	72.1	592.1	130.185	1.24616	81.65 ± 1.81	195.99 ± 96.98	203.59 ± 60.64
18	ethanol	C64175	C_2_H_6_O	46.1	486.5	95.38	1.04718	1817.75 ± 181.38 a	902.07 ± 104.80 b	686.32 ± 17.03 b
19	Acetone	C67641	C_3_H_6_O	58.1	506.8	101.254	1.11926	136.29 ± 6.92 b	489.36 ± 83.69 a	556.70 ± 90.59 a
20	3-Pentanone-M	C96220	C_5_H_10_O	86.1	695.1	177.393	1.11229	492.84 ± 36.29 a	172.45 ± 14.47 b	154.07 ± 25.53 b
21	3-Pentanone-D	C96220	C_5_H_10_O	86.1	695.8	177.893	1.35171	250.24 ± 28.43 a	95.32 ± 19.91 b	54.57 ± 12.07 b
22	Pentanal-M	C110623	C_5_H_10_O	86.1	696.2	178.143	1.19787	65.10 ± 1.73 c	447.41 ± 14.28 a	298.22 ± 5.20 b
23	Pentanal-D	C110623	C_5_H_10_O	86.1	696.9	178.642	1.42191	23.93 ± 2.11 b	264.06 ± 44.74 a	64.59 ± 8.66 b
24	Trimethylamine-M	C75503	C_3_H_9_N	59.1	560.1	118.495	0.95219	5685.93 ± 62.04 b	29,420.19 ± 3631.03 a	27,908.23 ± 2652.07 a
25	Trimethylamine-D	C75503	C_3_H_9_N	59.1	572.3	122.823	1.14403	379.96 ± 14.13 b	5247.13 ± 1364.67 a	3675.29 ± 643.97 a
26	benzaldehyde	C100527	C_7_H_6_O	106.1	975.4	506.648	1.14871	38.44 ± 4.78 b	183.86 ± 19.90 a	186.06 ± 26.67 a
27	Ammonia	C7664417	H_3_N	17.0	531.2	108.8	0.91403	48,938.49 ± 8308.36 b	67,713.77 ± 2317.18 a	76,358.79 ± 3022.60 a
28	3-hydroxy-2-butanone	C513860	C_4_H_8_O_2_	88.1	736.3	207.809	1.0711	700.48 ± 114.57 a	512.54 ± 34.65 b	706.27 ± 41.18 a

## Data Availability

Data is contained within the article.

## Supplementary Materials

The following supporting information can be downloaded at: https://www.mdpi.com/article/10.3390/foods11172710/s1, Table S1: Criteria for sensory evaluation; Table S2: Fatty acid content of hairtail under different frying conditions.

## References

[1] Luan L.L., Sun Y.S., Chen S.G., Wu C.H., Hu Y.Q. (2018). A study of fractal dimension as a quality indicator of hairtail (*Trichiurus haumela*) samples during frozen storage. Sci. Rep..

[2] Holdsworth S.D. (1997). Thermal Processing of Packaged Foods.

[3] Chumngoen W., Chen C.F., Tan F.J. (2018). Effects of moist-and dry-heat cooking on the meat quality, microstructure and sensory characteristics of native chicken meat. Anim. Sci. J..

[4] Wang S.Q., Chen H.T., Sun B.G. (2020). Recent progress in food flavor analysis using gas chromatography-ion mobility spectrometry (GC-IMS). Food Chem..

[5] Kumar V., Sharma H.K., Singh K., Kaushal P., Singh R.P. (2017). Effect of pre-frying drying on mass transfer kinetics of taro slices during deep fat frying. Int. Food Res. J..

[6] Zaghi A.N., Barbalho S.M., Guiguer E.L., Otoboni A.M. (2019). Frying Process: From Conventional to Air Frying Technology. Food Rev. Int..

[7] Larsen D., Quek S.Y., Eyres L. (2010). Effect of cooking method on the fatty acid profile of New Zealand King Salmon (*Oncorhynchus tshawytscha*). Food Chem..

[8] Shan J.H., Chen J.W., Xie D., Xia W.S., Xu W., Xiong Y.L.L. (2018). Effect of Xanthan Gum/Soybean Fiber Ratio in the Batter on Oil Absorption and Quality Attributes of Fried Breaded Fish Nuggets. J. Food Sci..

[9] Zhang W., Chen J.W., Yue Y., Zhu Z.Z., Liao E., Xia W.S. (2020). Modelling the Mass Transfer Kinetics of Battered and Breaded Fish Nuggets during Deep-Fat Frying at Different Frying Temperatures. J. Food Qual..

[10] Li X.R., Dong Y.F., Jiang P.F., Qi L.B., Lin S.Y. (2022). Identification of changes in volatile compounds in sea cucumber *Apostichopus japonicus* during seasonings soaking using HS-GC-IMS. LWT—Food Sci. Technol..

[11] Yao W.S., Cai Y.X., Liu D.Y., Chen Y., Li J.R., Zhang M.C., Chen N., Zhang H. (2022). Analysis of flavor formation during production of Dezhou braised chicken using headspace-gas chromatography-ion mobility spec-trometry (HS-GC-IMS). Food Chem..

[12] Song J.X., Shao Y., Yan Y.M., Li X.H., Peng J., Guo L. (2021). Characterization of volatile profiles of three colored quinoas based on GC-IMS and PCA. LWT—Food Sci. Technol..

[13] Wang D., Zhang J., Zhu Z.S., Lei Y., Huang S.H., Huang M. (2022). Effect of ageing time on the flavour compounds in Nanjing water-boiled salted duck detected by HS-GC-IMS. LWT—Food Sci. Technol..

[14] AOAC International (2004). Official Methods of Analysis.

[15] Wang Y., Jiang Y.T., Cao J.X., Chen Y.J., Sun Y.Y., Zeng X.Q., Pan D.D., Ou C.R., Gan N. (2016). Study on lipolysis-oxidation and volatile flavour compounds of dry-cured goose with different curing salt content during production. Food Chem..

[16] Hu X.F., Li J.L., Zhang L., Wang H., Peng B., Hu Y.M., Liang Q.X., Tu Z.C. (2021). Effect of frying on the lipid oxidation and volatile substances in grass carp (*Ctenopharyngodon idellus*) fillet. J. Food Process. Preserv..

[17] Isik B., Sahin S., Sumnu G. (2016). Pore Development, Oil and Moisture Distribution in Crust and Core Regions of Potatoes during Frying. Food Bioprocess. Technol..

[18] Yu X.N., Li L.Q., Xue J., Wang J., Song G.S., Zhang Y.Q., Shen Q. (2020). Effect of air-frying conditions on the quality attributes and lipidomic characteristics of surimi during processing. Innov. Food Sci. Emerg..

[19] Fang M.C., Huang G.J., Sung W.C. (2021). Mass transfer and texture characteristics of fish skin during deep-fat frying, electrostatic frying, air frying and vacuum frying. LWT—Food Sci. Technol..

[20] Li Y.Q., Li C.B., Zhao F., Lin X.S., Bai Y., Zhou G.H. (2016). The Effects of Long-Duration Stewing Combined with Different Cooking and Heating Methods on the Quality of Pork Belly. J. Food Process. Preserv..

[21] Cao Y., Wu G.C., Zhang F., Xu L.R., Jin Q.Z., Huang J.H., Wang X.G. (2020). A Comparative Study of Physicochemical and Flavor Characteristics of Chicken Nuggets during Air Frying and Deep Frying. J. Am. Oil Chem. Soc..

[22] Zeng H., Chen J.W., Zhai J.L., Wang H.B., Xia W.S., Xiong Y.L.L. (2016). Reduction of the fat content of battered and breaded fish balls during deep-fat frying using fermented bamboo shoot dietary fiber. LWT—Food Sci. Technol..

[23] Yu M., He S.D., Tang M.M., Zhang Z.Y., Zhu Y.S., Sun H.J. (2018). Antioxidant activity and sensory characteristics of Maillard reaction products derived from different peptide fractions of soybean meal hydrolysate. Food Chem..

[24] Vieira E.C.S., Marsico E.T., Conte C.A., Damiani C., Canto A., Monteiro M.L.G., da Silva F.A. (2018). Effects of different frying techniques on the color, fatty acid profile, and lipid oxidation of *Arapaima gigas*. J. Food Process. Preserv..

[25] Pedreschi F., Moyano P., Kaack K., Granby K. (2005). Color changes and acrylamide formation in fried potato slices. Food Res. Int..

[26] Jiang S., Xue D.J., Zhang Z., Shan K., Ke W.X., Zhang M., Zhao D., Nian Y.Q., Xu X.L., Zhou G.H. (2022). Effect of Sous-vide cooking on the quality and digestion characteristics of braised pork. Food Chem..

[27] Wu R.L., Jiang Y., Qin R.K., Shi H.N., Jia C.H., Rong J.H., Liu R. (2022). Study of the formation of food hazard factors in fried fish nuggets. Food Chem..

[28] Roldan M., Antequera T., Armenteros M., Ruiz J. (2014). Effect of different temperature-time combinations on lipid and protein oxidation of sous-vide cooked lamb loins. Food Chem..

[29] Luo X.Y., Xiao S.T., Ruan Q.F., Gao Q., An Y.Q., Hu Y., Xiong S.B. (2022). Differences in flavor characteristics of frozen surimi products reheated by microwave, water boiling, steaming, and frying. Food Chem..

[30] Pimentel T.C., Madrona G.S., Prudencio S.H. (2015). Probiotic clarified apple juice with oligofructose or sucralose as sugar substitutes: Sensory profile and acceptability. LWT—Food Sci. Technol..

[31] Giri A., Osako K., Ohshima T. (2010). Identification and characterisation of headspace volatiles of fish *miso*, a Japanese fish meat based fermented paste, with special emphasis on effect of fish species and meat washing. Food Chem..

[32] Ding A.Z., Zhu M., Qian X.Q., Shi L., Huang H., Xiong G.Q., Wang J., Wang L. (2020). Effect of fatty acids on the flavor formation of fish sauce. LWT—Food Sci. Technol..

[33] Nayak P.K., Dash U., Rayaguru K., Krishnan K.R. (2016). Physio-Chemical Changes During Repeated Frying of Cooked Oil: A Review. J. Food Biochem..

[34] Na W., Xi C. (2019). Identification of important odorants derived from phosphatidylethanolamine species in steamed male *Eriocheir sinensis* hepatopancreas in model systems. Food Chem..

[35] Zhou Y., Wu S., Peng Y., Jin Y., Xu X. (2021). Effect of Lactic Acid Bacteria on Mackerel (*Pneumatophorus japonicus*) Seasoning Quality and Flavor during Fermentation. Food Biosci..

[36] Tanimoto S., Kitabayashi K., Fukusima C., Sugiyama S., Hashimoto T. (2015). Effect of storage period before reheating on the volatile compound composition and lipid oxidation of steamed meat of yellowtail *Seriola quinqueradiata*. Fish. Sci..

[37] Watanabe A., Kamada G., Imanari M., Shiba N., Yonai M., Muramoto T. (2015). Effect of aging on volatile compounds in cooked beef. Meat Sci..

[38] An Y., Qian Y.L., Alcazar Magana A., Xiong S., Qian M.C. (2020). Comparative Characterization of Aroma Compounds in Silver Carp (*Hypophthalmichthys molitrix*), Pacific Whiting (*Merluccius productus*), and Alaska Pollock (*Theragra chalcogramma*) Surimi by Aroma Extract Dilution Analysis, Odor Activity Value, and Aroma Recombination Studies. J. Agric. Food Chem..

[39] Yi C., Pla B., Lla B., Yq C., Lja B., Yang L.A. (2021). Characteristic fingerprints and volatile flavor compound variations in Liuyang Douchi during fermentation via HS-GC-IMS and HS-SPME-GC-MS. Food Chem..

[40] Jeleń H., Gracka A. (2016). Characterization of aroma compounds: Structure, physicohemical and sensory properties. Flavour: From Food to Perception.

[41] Fernández de Palencia P., de la Plaza M., Mohedano M.L., Martínez-Cuesta M.C., Requena T., López P., Peláez C. (2004). Enhancement of 2-methylbutanal formation in cheese by using a fluorescently tagged Lacticin 3147 producing *Lactococcus lactis* strain. Int. J. Food Microbiol..

[42] Yu H.Y., Xie T., Xie J.R., Chen C., Ai L.Z., Tian H.X. (2020). Aroma perceptual interactions of benzaldehyde, furfural, and vanillin and their effects on the descriptor intensities of *Huangjiu*. Food Res. Int..

[43] Salum P., Guclu G., Selli S. (2017). Comparative Evaluation of Key Aroma-Active Compounds in Raw and Cooked Red Mullet (*Mullus barbatus*) by Aroma Extract Dilution Analysis. J. Agric. Food Chem..

[44] Duan Z.L., Dong S.L., Sun Y.X., Dong Y.W., Gao Q.F. (2021). Response of Atlantic salmon (*Salmo salar*) flavor to environmental salinity while culturing between freshwater and seawater. Aquaculture.

[45] Zhang L., Hu Y.Y., Wang Y., Kong B.H., Chen Q. (2021). Evaluation of the flavour properties of cooked chicken drumsticks as affected by sugar smoking times using an electronic nose, electronic tongue, and HS-SPME/GC-MS. LWT—Food Sci. Technol..

[46] Madruga M.S., Elmore J.S., Oruna-Concha M.J., Balagiannis D., Mottram D.S. (2010). Determination of some water-soluble aroma precursors in goat meat and their enrolment on flavour profile of goat meat. Food Chem..

[47] Mathieu S., Cin V.D., Zhang J.F., Hua L., Bliss P., Mark G.T., Klee H.J., Tieman D.M. (2009). Flavour compounds in tomato fruits: Identification of loci and potential pathways affecting volatile composition. J. Exp. Bot..

[48] Longo M.A., Sanromán M.A. (2005). Production of Food Aroma Compounds: Microbial and Enzymatic Methodologies. Food Technol. Biotech..

[49] Liu D.Y., Bai L., Feng X., Chen Y.P., Zhang D.N., Yao W.S., Zhang H., Chen G.L., Liu Y. (2020). Characterization of *Jinhua ham* aroma profiles in specific to aging time by gas chromatography-ion mobility spectrometry (GC-IMS). Meat Sci..

[50] Sebzalli Y.M., Wang X.Z. (2001). Knowledge discovery from process operational data using PCA and fuzzy clustering. Eng. Appl. Artif. Intel..

[51] Song J.X., Yan Y.M., Wang X.D., Li X.H., Chen Y., Li L., Li W.H. (2021). Characterization of fatty acids, amino acids and organic acids in three colored quinoas based on untargeted and targeted metabolomics. LWT—Food Sci. Technol..

[52] Lorenzo J.M., Carballo J., Franco D. (2014). Effect of the inclusion of chestnut in the finishing diet on volatile compounds during the manufacture of dry-cured “Lacon” from Celta pig breed. Meat Sci..

[53] Xia C., He Y., Cheng S., He J., Sun Y. (2020). Free fatty acids responsible for characteristic aroma in various sauced-ducks. Food Chem..

[54] Ba H.V., Seo H.W., Seong P.N., Cho S.H., Kang S.M., Kim Y.S., Moon S.S., Choi Y.M., Kim J.H. (2019). Live weights at slaughter significantly affect the meat quality and flavor components of pork meat. Anim. Sci. J..

