# Supplementary material for: Comparative Studies on the Physicochemical and Volatile Flavour Properties of Traditional Deep Fried and Circulating-Air Fried Hairtail (Trichiurus lepturus)
Source: Foods. 2022 Sep 5;11(17):2710. doi: 10.3390/foods11172710 (PMC9455639; doi:10.3390/foods11172710)
Supplement: Supplementary file 1 [file foods-11-02710-s001.zip › foods-1848452-supplementary.pdf]

Supplementary materials

Table S1. Criteria for sensory evaluation.

Project.	Standard description	Score
Color	Red brown or burnt yellow in color, well-distributed	8-10
	Light yellow in color, less well-distributed	5-7
	The color is light and not distributed enough	1-4
Tissue	The fish is firm and crispy and complete	8-10
	The fish is less firm, brittle and less damaged	5-7
	Fish is loose, not crisp and badly damaged	1-4
Odor	the characteristic flavor of fried hairtail and no peculiar smell	8-10
	characteristics of slightly worse flavor of fried hairtail, slightly fishy smell, slightly burnt, bitter and other odor	5-7
	no fried hairtail flavor, fishy heavy, burnt, bitter and other heavy	1-4
Taste	tender, soft and hard, and chewy	8-10
	more tender, soft or hard, and not chewy enough	5-7
	rough, too soft or too hard, and not chewy	1-4
Overall Acceptability	Easy to accept	8-10
	Can be accept	5-7
	unacceptable	1-4

Table S2. Fatty acid content of fish under different frying conditions. g/100g.

	Fatty acid	Raw	DF hairtail	AF hairtail
SFA	C14:0	0.0045±0.0016[b]	0.0190±0.0019[a]	0.0094±0.0023[b]
	C15:0	0.0013±0.0002[a]	0.0030±0.0001[a]	0.0009±0.0000[a]
	C16:0	0.0779±0.0120[c]	1.9863±0.0209[a]	0.1645±0.0139[b]
	C17:0	0.0019±0.0006[c]	0.0213±0.0002[a]	0.0053±0.0010[b]
	C18:0	0.0375±0.0062[c]	0.9066±0.0093[a]	0.0878±0.0107[b]
	C20:0	-	0.0834±0.0010	-
	C22:0	0.0016±0.0006[b]	0.0977±0.0010[a]	0.0018±0.0000[b]
	C23:0	-	0.0117±0.0000	-
	C24:0	0.0015±0.0003[b]	0.0425±0.0009[a]	0.0038±0.0003[b]
MUFA	C16:1	0.0051±0.0004[c]	0.0222±0.0011[a]	0.0118±0.0016[b]
	C18:1n9c	0.0700±0.0232[b]	4.0265±0.0215[a]	0.1132±0.0106[b]
	C20:1	0.0035±0.0006[b]	0.0376±0.0001[a]	0.0086±0.0024[b]
	C22:1n9	0.0121±0.0006[b]	0.0287±0.0016[a]	0.0358±0.0026[a]
	C24:1	0.0067±0.0007[b]	0.0180±0.0001[a]	0.0186±0.0028[a]
PUFA	C18:2n6c	0.0761±0.0457[b]	8.2726±0.0554[a]	0.0300±0.0149[b]
	C18:3n3	0.0091±0.0051[b]	0.8587±0.0084[a]	0.0101±0.0018[b]
	C20:2	0.0011±0.0000[c]	0.0104±0.0000[a]	0.0031±0.0006[b]
	C20:3n3	0.0019±0.0006[a]	0.0027±0.0005[a]	0.0066±0.0039[a]
	C20:4n6	0.0216±0.0012[a]	0.0256±0.0016[a]	0.0247±0.0042[a]
	EPA C20:5n3	0.0121±0.0011[b]	0.0223±0.0032[a]	0.0297±0.0011[a]
	DHA C22:6n3	0.0918±0.0066[c]	0.1813±0.0034[b]	0.2585±0.0153[a]

The different letters in the same column represent a significant difference with $p < 0.05$ among each group.
